# Supplementary material for: Gut microbiome alterations among Ghanaian children with asymptomatic malaria infections
Source: PLoS One. 2026 Apr 24;21(4):e0348120. doi: 10.1371/journal.pone.0348120 (PMC13108879; doi:10.1371/journal.pone.0348120)
Supplement: Attachment — Submitted filename: PONE-D-26-02351 REV.pdf [file pone.0348120.s009.pdf]

PLOS One

# Gut microbiome alterations among Ghanaian children with asymptomatic malaria infections

--Manuscript Draft--

| | |
|---|---|
| **Manuscript Number:** | PONE-D-26-02351 |
| **Article Type:** | Research Article |
| **Full Title:** | Gut microbiome alterations among Ghanaian children with asymptomatic malaria infections |
| **Short Title:** | Asymptomatic malaria and the gut microbiome |
| **Corresponding Author:** | Amma Aboagyewa Larbi, BSc, MSc, PhD<br>Kwame Nkrumah University of Science and Technology<br>Kumasi, GHANA |
| **Keywords:** | Malaria, Gut Microbiome, Alpha Diversity, Beta Diversity, Microbial Dysbiosis, Plasmodium |
| **Abstract:** | The human gut microbiome, consisting of bacteria, archaea, fungi, and viruses, influences various physiological processes of the body. The gut microbiome composition is shaped by factors such as diet, geography, and antibiotic use. Malaria has been a global health challenge over the years, especially in low- and middle-income countries. This study investigated how asymptomatic malaria infection altered gut microbial communities in Ghanaian children, offering insights for novel malaria control strategies. Standard aseptic phlebotomy procedures were employed to collect venous blood samples for Plasmodium species detection through microscopy, rapid diagnostic testing (RDT), and real-time PCR. The gut microbial community was profiled by sequencing the 16S rRNA V4 region, and sequence data were processed using the DADA2 pipeline in R. Taxonomic classification was assigned with high confidence using the SILVA v138 reference database. Asymptomatic malaria infections were predominantly mixed with P. falciparum and P. malariae. Microbiome analysis revealed that Firmicutes and Bacteroidetes comprised nearly 70% of the total microbial population. Asymptomatic individuals showed a decrease in Firmicutes and an increase in Bacteroidetes, with a slight increase in Proteobacteria. At the genus level, Prevotella_9 was the most abundant and exhibited the highest variability in the infected groups. The Alloprevotella and Streptococcus genera increased in both infected groups, but Escherichia-Shigella was elevated in only those with mixed infections. Faecalibacterium declined in asymptomatic malaria-infected individuals compared to healthy controls, with variability further reduced in mixed infections. Beta-diversity analysis indicated a significant effect of malaria status on microbial composition (PERMANOVA, $p < 0.05$), explaining approximately 19.1% of the total variation captured by a 2D PCA projection. This confirms that infection status significantly influenced gut microbial community structure. These findings suggest a potential link between malaria infection and gut microbiota alterations and highlight microbial shifts associated with disease status. |
| **Order of Authors:** | Amma Aboagyewa Larbi, BSc, MSc, PhD |
| | Moses Etsey, BSc |
| | Obed Brew |
| | Bismark Koduah |
| | Rosemond Enam Mawuenyega |
| | Emmanuel Kobla Atsu Amewu |
| | Nehemiah Kweku Essilfie |
| | Solomon Wireko |
| | Alexander Kwarteng |
| | Ben Adu Gyan |

Powered by Editorial Manager® and ProduXion Manager® from Aries Systems Corporation

| Additional Information: | |
|---|---|
| **Question** | **Response** |
| **Financial Disclosure**<br><br>Enter a financial disclosure statement that describes the sources of funding for the work included in this submission. Review the submission guidelines for detailed requirements. View published research articles from *PLOS ONE* for specific examples.<br><br>This statement is required for submission and **will appear in the published article** if the submission is accepted. Please make sure it is accurate.<br><br>**Funded studies**<br>Enter a statement with the following details:<br>• Initials of the authors who received each award<br>• Grant numbers awarded to each author<br>• The full name of each funder<br>• URL of each funder website<br>• Did the sponsors or funders play any role in the study design, data collection and analysis, decision to publish, or preparation of the manuscript?<br><br>Did you receive funding for this work? | The author(s) received no specific funding for this work. |
| **Competing Interests**<br><br>Use the instructions below to enter a competing interest statement for this submission. On behalf of all authors, disclose any competing interests that could be perceived to bias this work—acknowledging all financial support and any other relevant financial or non-financial competing interests.<br><br>This statement is required for submission and **will appear in the published article** if the submission is accepted. Please make sure it is accurate and that any funding sources listed in your Funding Information | The authors have declared that no competing interests exist. |

| | |
|---|---|
| later in the submission form are also declared in your Financial Disclosure statement.<br><br>View published research articles from *PLOS ONE* for specific examples.<br><br>**NO authors have competing interests**<br><br>Enter: *The authors have declared that no competing interests exist.*<br><br>**Authors with competing interests**<br><br>Enter competing interest details beginning with this statement:<br><br>*I have read the journal's policy and the authors of this manuscript have the following competing interests: [insert competing interests here]*<br><br>* typeset | |
| **Ethics Statement**<br><br>Enter an ethics statement for this submission. This statement is required if the study involved:<br><br>• Human participants<br>• Human specimens or tissue<br>• Vertebrate animals or cephalopods<br>• Vertebrate embryos or tissues<br>• Field research<br><br>Write "N/A" if the submission does not require an ethics statement.<br><br>General guidance is provided below. Consult the submission guidelines for detailed instructions. **Make sure that all information entered here is included in the Methods section of the manuscript.** | This study was approved by the Committee on Human Research, Publication, and Ethics (CHRPE) at the Kwame Nkrumah University of Science and Technology (KNUST) and Komfo Anokye Teaching Hospital, Kumasi (Approval No.CHRPE/AP/190/22). Additional authorisation was obtained from the Ahanta West Municipal Health Directorate. All procedures involving human participants were conducted in accordance with the ethical principles outlined in the Declaration of Helsinki. Written informed consent was obtained from parents or legal guardians of all participating children prior to sample collection. |

Powered by Editorial Manager® and ProduXion Manager® from Aries Systems Corporation

| | |
|---|---|
| **Format for specific study types**<br><br>**Human Subject Research (involving human participants and/or tissue)**<br>• Give the name of the institutional review board or ethics committee that approved the study<br>• Include the approval number and/or a statement indicating approval of this research<br>• Indicate the form of consent obtained (written/oral) or the reason that consent was not obtained (e.g. the data were analyzed anonymously)<br><br>**Animal Research (involving vertebrate animals, embryos or tissues)**<br>• Provide the name of the Institutional Animal Care and Use Committee (IACUC) or other relevant ethics board that reviewed the study protocol, and indicate whether they approved this research or granted a formal waiver of ethical approval<br>• Include an approval number if one was obtained<br>• If the study involved *non-human primates*, add *additional details* about animal welfare and steps taken to ameliorate suffering<br>• If anesthesia, euthanasia, or any kind of animal sacrifice is part of the study, include briefly which substances and/or methods were applied<br><br>**Field Research**<br><br>Include the following details if this study involves the collection of plant, animal, or other materials from a natural setting:<br>• Field permit number<br>• Name of the institution or relevant body that granted permission | |
| **Data Availability**<br><br>Authors are required to make all data underlying the findings described fully available, without restriction, and from the time of publication. PLOS allows rare exceptions to address legal and ethical concerns. See the PLOS Data Policy and FAQ for detailed information. | Yes - all data are fully available without restriction |

| | |
|---|---|
| A Data Availability Statement describing where the data can be found is required at submission. Your answers to this question constitute the Data Availability Statement and **will be published in the article**, if accepted.<br><br>**Important:** Stating 'data available on request from the author' is not sufficient. If your data are only available upon request, select 'No' for the first question and explain your exceptional situation in the text box.<br><br>Do the authors confirm that all data underlying the findings described in their manuscript are fully available without restriction? | |
| **Describe where the data may be found in full sentences. If you are copying our sample text, replace any instances of XXX with the appropriate details.**<br><br>• If the data are **held or will be held in a public repository**, include URLs, accession numbers or DOIs. If this information will only be available after acceptance, indicate this by ticking the box below. For example: *All XXX files are available from the XXX database (accession number(s) XXX, XXX.).*<br>• If the data are all contained **within the manuscript and/or Supporting Information files**, enter the following: *All relevant data are within the manuscript and its Supporting Information files.*<br>• If neither of these applies but you are able to provide **details of access elsewhere**, with or without limitations, please do so. For example:<br><br>*Data cannot be shared publicly because of [XXX]. Data are available from the XXX Institutional Data Access / Ethics Committee (contact via XXX) for researchers who meet the criteria for access to confidential data.*<br><br>*The data underlying the results presented in the study are available from (include the name of the third party* | All relevant data are within the manuscript and its Supporting Information files. |

| | |
|---|---|
| *and contact information or URL).*<br>• This text is appropriate if the data are owned by a third party and authors do not have permission to share the data.<br><br>* typeset | |
| Additional data availability information: | |

Manuscript

Click here to access/download;Manuscript;Main Microbiome_Malaria Manuscript.docx

# Gut microbiome alterations among Ghanaian children with asymptomatic malaria infections

Amma Aboagyewa Larbi[1], Moses Etsey[1], Obed Brew[2], Bismark Koduah[1], Rosemond Enam Mawuenyega[1], Emmanuel Kobla Atsu Amewu[1], Nehemiah Kweku Essilfie[1], Solomon Wireko[4], Alexander Kwarteng[1,3], Ben Adu Gyan[5].

[1]Department of Biochemistry and Biotechnology, College of Science, Kwame Nkrumah University of Science and Technology, Kumasi, Ghana.

[2]Department of Pharmacology, College of Health Sciences, Kwame Nkrumah University of Science and Technology, Kumasi, Ghana.

[3]Kumasi Centre for Collaborative Research in Tropical Medicine, Kumasi, Ghana.

[4]Kumasi Technical University, Kumasi, Ghana.

[5]Noguchi Memorial Institute for Medical Research, University of Ghana, Legon, Ghana.

**Corresponding Author:**

E-mail: ammalarbi@gmail.com (AAL)

# Abstract

The human gut microbiome, consisting of bacteria, archaea, fungi, and viruses, influences various physiological processes of the body. The gut microbiome composition is shaped by factors such as diet, geography, and antibiotic use. Malaria has been a global health challenge over the years, especially in low- and middle-income countries. This study investigated how asymptomatic malaria infection altered gut microbial communities in Ghanaian children, offering insights for novel malaria control strategies. Standard aseptic phlebotomy procedures were employed to collect venous blood samples for Plasmodium species detection through microscopy, rapid diagnostic testing (RDT), and real-time PCR. The gut microbial community was profiled by sequencing the 16S rRNA V4 region, and sequence data were processed using the DADA2 pipeline in R. Taxonomic classification was assigned with high confidence using the SILVA v138 reference database. Asymptomatic malaria infections were predominantly mixed with P. falciparum and P. malariae. Microbiome analysis revealed that Firmicutes and Bacteroidetes comprised nearly 70% of the total microbial population. Asymptomatic individuals showed a decrease in Firmicutes and an increase in Bacteroidetes, with a slight increase in Proteobacteria. At the genus level, Prevotella_9 was the most abundant and exhibited the highest variability in the infected groups. The Alloprevotella and Streptococcus genera increased in both infected groups, but Escherichia-Shigella was elevated in only those with mixed infections. Faecalibacterium declined in asymptomatic malaria-infected individuals compared to healthy controls, with variability further reduced in mixed infections. Beta-diversity analysis indicated a significant effect of malaria status on microbial composition (PERMANOVA, $p < 0.05$), explaining approximately 19.1% of the total variation captured by a 2D PCA projection. This confirms that infection status significantly influenced gut microbial community structure. These findings suggest a potential link between malaria infection and gut microbiota alterations and highlight microbial shifts associated with disease status.

**Keywords**: Malaria, Gut Microbiome, Alpha Diversity, Beta Diversity, Microbial Dysbiosis, *Plasmodium*

# Introduction

Malaria remains a major health challenge in sub-Saharan Africa, where Plasmodium infections are highly endemic and disproportionately affect children. Asymptomatic malaria infections play a critical role in sustaining transmission and maintaining parasite reservoirs within endemic communities (1). These silent carriers act as hidden reservoirs that complicate malaria control and elimination efforts (1). Beyond its direct clinical impact, malaria infection also alters host physiology, including gut microbial dysbiosis, immune and metabolic pathways (2,3). The interaction between malaria infection and the gut microbiome has been reported to play a role in malaria pathogenesis and influence host immunity and disease severity (4). The human gut microbiome consists of diverse microorganisms (bacteria, archaea, fungi, and viruses) that influence various physiological processes of the body (5). The composition of the gut microbiome is shaped by factors that impact health and disease and some of these factors include diet, geography, genetics, infection, and antibiotic use (3).

The disruption of gut microbial balance has been observed in malaria infections. Shifts in the abundance of Firmicutes, Bacteroidetes, and Proteobacteria have been reported and have potential consequences for intestinal integrity and systemic inflammation (6). The dominant microbial phyla in the gut that contribute to the metabolism of nutrients, immune homeostasis of the body, and susceptibility to other infections are Firmicutes and Bacteroidetes (7,8). Firmicutes is a phylum involved in gut homeostasis and short-chain fatty acid production, while Proteobacteria are linked to inflammation. As a result, a decrease in Firmicutes and an increase in Proteobacteria have been

noted in malaria-infected individuals, suggesting that malaria can disrupt gut microbial stability (9,10). This emerging evidence suggests that these alterations have broader implications beyond the infection itself and can alter metabolic pathways that will have potential consequences on recovery from the infection and response to treatment.

Despite emerging evidence linking the microbiome to malaria pathogenesis, most studies have focused on symptomatic cases, leaving limited understanding of asymptomatic infections in African children (11). Research to understand these microbiome-related changes in asymptomatic malaria cases holds the potential to provide new insights into host-pathogen interactions and pave the way for microbiome-targeted interventions to complement the existing malaria control strategies. As a result, this study aims to characterize the gut microbiome composition in Ghanaian children with asymptomatic malaria to determine microbial shifts associated with asymptomatic *Plasmodium* infections.

# Materials and methods

## Ethical statement

This study was conducted in accordance with the Declaration of Helsinki and approved by the Committee on Human Research, Publication, and Ethics (CHRPE) at the Kwame Nkrumah University of Science and Technology (KNUST) and Komfo Anokye Teaching Hospital, Kumasi (Approval No. CHRPE/AP/190/22). Additional authorisation was obtained from the Ahanta West Municipal Health Directorate. Written informed consent was obtained from parents or legal guardians of all participating children prior to sample collection.

## Study design and study site

A cross-sectional design was employed, involving 113 school-aged children (6–15 years) residing in the Ahanta West Municipality, Western Region, Ghana - an area of perennial malaria transmission. Participants were prospectively recruited between December 4, 2022 and December 30, 2022 through community health screenings. Inclusion criteria required asymptomatic status (absence of fever or malaria symptoms at the time of sampling) and residence within the study area. Children who had taken antibiotics or antimalarial medication within two weeks prior to sampling were excluded.

## Sample collection

Venous blood samples (2–3 mL) were collected aseptically into EDTA tubes using standard phlebotomy procedures for malaria diagnosis. Stool samples were obtained using sterile containers prefilled with 2 mL RNAlater (Invitrogen, USA) to stabilise microbial RNA. Each sample was labelled with a unique identifier and stored on ice during transport to the laboratory, then frozen at –20°C until processing.

## Malaria diagnosis

Thick and thin blood smears were prepared and stained with 10% Giemsa for parasite detection and quantification under 100× oil immersion microscopy. Rapid diagnostic tests (RDTs) were conducted using Bioline™ Malaria Ag P.f/Pan test kits according to manufacturer instructions. Molecular confirmation and Plasmodium species identification were performed using the Novaplex™ Malaria Assay (Seegene, Korea) on a Bio-Rad CFX Maestro real-time PCR system. Species-specific fluorescence signals enabled differentiation of P. falciparum, P. malariae, and P. ovale. Positive and negative controls were included in each batch.

## DNA extraction and 16S rRNA gene sequencing

Microbial DNA was extracted from stool samples using the QIAamp DNA Stool Mini Kit (Qiagen, Germany) following the manufacturer’s instructions, with extraction blanks included as negative controls. DNA yield and purity were assessed using a NanoDrop spectrophotometer and Qubit 3.0 fluorometer. The V4 hypervariable region of the bacterial 16S rRNA gene was amplified using universal primers 515F/806R and Platinum Hot Start PCR Master Mix (Thermo Fisher Scientific). PCR products were purified, normalised, and pooled equimolarly prior to sequencing on an Illumina MiSeq platform (MiSeq Reagent Kit v3; 2×270 bp paired-end reads). Library quality was confirmed via capillary electrophoresis, and PhiX (15–20%) was spiked in to improve base diversity.

## Bioinformatics and statistical analysis

Demultiplexed paired-end reads were processed in R (v4.3.3) using the DADA2 pipeline for quality filtering, error correction, merging, and chimera removal, generating an amplicon sequence variant (ASV) table. Taxonomic classification was performed using the SILVA v138 reference database and the naïve Bayesian classifier. The resulting ASV, taxonomy, and metadata tables were integrated in Phyloseq for diversity analyses. Low-prevalence ASVs were filtered (<0.01% of total reads). Alpha diversity (Observed Richness and Shannon index) was computed after rarefaction, and between-group differences were tested using the Wilcoxon rank-sum test. Beta diversity was assessed using Aitchison distance and visualised via Principal Component Analysis (PCA), with statistical significance evaluated using PERMANOVA (999 permutations). Differential abundance analysis was conducted in DESeq2, with multiple testing correction applied (Benjamini–Hochberg FDR < 0.05). Data visualisation was performed with ggplot2 (v3.4.4).

# Results

## Demographic characteristics of participants

One hundred and thirteen (113) children were recruited for the study, with more than half being females (61.9%). The distribution of the demographics of the study participants is presented in Table 1.

**Table 1: Age, height, and weight distribution of study participants**

| Parameter | Median | Interquartile Range |
|---|---|---|
| Age | 12 | (10.0 - 14.0) |
| Height (cm) | 140.2 | (132.0 - 152.1) |
| Weight (kg) | 32.1 | (26.0 - 42.8) |

## Distribution of asymptomatic malaria infections

From Fig 1, mixed infections were more prevalent among the study participants, with the majority harbouring both *P. falciparum* and *P. malariae*.

**Fig 1: Distribution of asymptomatic malaria infections**

## Microbiome dynamics in asymptomatic malaria patients (*P. falciparum* only)

### Prevalence of microbial phyla

In this study, twelve microbial phyla were identified with Firmicutes and Bacteroidetes accounting for almost 70% of the total microbial population. Fig 2 illustrates the prevalence of different microbial phyla across samples. This figure represents the relative abundance – the proportional representation of each bacterial phylum within samples. The plot shows the composition of bacterial phyla in stool samples from healthy controls and individuals with asymptomatic *Plasmodium falciparum*-only infection. Firmicutes and Bacteroidetes dominated the gut microbiota in both groups. Compared with healthy participants, the *P. falciparum*-only group exhibited a modest decrease in Firmicutes and an increase in Bacteroidetes, along with a slight rise in Proteobacteria.

**Fig 2: Relative abundance of bacterial phyla in asymptomatic *P. falciparum*-only infection**

### Relative abundance of microbial taxa

Fig 3 and 4 illustrates the abundance of the microbial taxa and distribution at the genus level for individuals infected with *P. falciparum* only relative to healthy group. Relative abundance of dominant bacterial genera among healthy controls and individuals with asymptomatic P. falciparum-only infection. Prevotella_9 was the most abundant genus, followed by Faecalibacterium and Blautia. Compared with healthy controls, asymptomatic malaria cases showed a reduction in Faecalibacterium and increases in Alloprevotella and Streptococcus, indicating early microbial

imbalance marked by the loss of beneficial butyrate producers and enrichment of pro-inflammatory taxa.

**Fig 3: Genus-level distribution in asymptomatic *P. falciparum*-only infection**

Fig 4 shows the relative prevalence of dominant bacterial phyla identified in stool samples from healthy controls and individuals with asymptomatic *P~~lasmodium~~ falciparum* infection. Firmicutes and Bacteroidetes accounted for roughly 70 % of total taxa.

**Figure 4: Prevalence of microbial phyla in asymptomatic *P. falciparum*-only infection**

Axes/Legend labels: x-axis: Participant group (Healthy | *P. falciparum*); y-axis: Relative prevalence (%).

## Microbial diversity

Shannon diversity index analysis was performed to assess and compare microbial species richness and evenness between healthy individuals and those with asymptomatic *P. falciparum*-only infections, Fig 5. Although the difference indicates a marginal increase in microbial diversity among malaria cases, it was not statistically significant ($p > 0.05$). The substantial overlap in diversity ranges rather suggests that overall gut microbial richness and evenness remain largely stable despite infection. The presence of outliers in both groups further highlights pronounced inter-individual variability in gut microbial composition. The malaria group exhibited a slightly higher median Shannon index (3.8, IQR: 3.5–4.1) compared with the healthy group (3.6, IQR: 3.2–4.0), although the difference was not statistically significant ($p > 0.05$). The substantial overlap in interquartile ranges suggests that overall gut microbial richness and evenness remained largely stable despite infection.

**Fig 5: Shannon diversity index comparing healthy and *P. falciparum*-only infections**

## Microbiome dynamics in asymptomatic malaria patients (Mixed infection)

### Prevalence of microbial phyla

A total of twelve microbial phyla were identified in the asymptomatic malaria group with mixed infection involving *P. falciparum* and *P. malariae,* Fig 6. The overall phylum-level composition mirrored that of the single-infection group, Fig 3, with Firmicutes and Bacteroidetes remaining dominant. However, subtle shifts in relative abundance were observed, particularly a modest decrease in Bacteroidetes and a compensatory increase in Firmicutes. These variations suggest that mixed-species Plasmodium infections may exert distinct selective pressures on gut microbial communities.

**Fig 6: Prevalence of microbial phyla in mixed *P. falciparum* and *P. malariae* infections**

Relative prevalence of microbial phyla among healthy controls and individuals harbouring mixed asymptomatic *P. falciparum* and *P. malariae* infections. Axes/Legend labels: x-axis: Participant group (Healthy | Mixed infection); y-axis: Relative prevalence (%).

### Relative abundance of microbial taxa

In asymptomatic malaria-infected individuals with mixed infection, Bacteroidetes and Firmicutes were highly abundant in both groups, as shown in Fig 7. Compared to the control group, Firmicutes

decreased in asymptomatic malaria-infected individuals with mixed infection, while Bacteroidetes increased. Proteobacteria also showed a slight increase in the asymptomatic malaria-infected group. When compared to asymptomatic malaria-infected individuals with *P. falciparum* only, the mixed infection group exhibited a reduction in Bacteroidetes and an increase in Firmicutes, with less variability. The abundance of Spirochaetota remained relatively constant but showed reduced variability. These trends indicate that co-infection exerts additional but mild selective pressures on gut microbial composition.

**Fig 7: Relative abundance of bacterial phyla in mixed *P. falciparum* and *P. malariae* infections**

Fig 8 shows that the *Prevotella_9* genus exhibited the highest variability and was the most abundant in both groups, with an increase in the infected group. The abundance of *Alloprevotella* increased in asymptomatic malaria-infected individuals with mixed infection compared to healthy controls. *Faecalibacterium* was the second most abundant genus in the healthy group but showed a decline in the asymptomatic malaria-infected group, with reduced variability. Its variability was also lower compared to individuals with *P. falciparum* only. Compared to asymptomatic malaria-infected individuals with *P. falciparum* only, the mixed infection group exhibited a reduction in *Prevotella_9* and *Streptococcus*, while *Escherichia-Shigella* showed an increase in abundance. These patterns suggest that co-infection amplifies microbial compositional shifts, potentially heightening inflammation-related microbial signatures.

**Fig 8: Genus-level distribution in mixed *P. falciparum* and *P. malariae* infections**

## Microbial diversity

Fig 9 presents the Shannon diversity index comparing healthy individuals with those harbouring mixed P. falciparumand P. malariae infections. The malaria group exhibited a slightly higher median Shannon index (3.7, IQR: 3.4–4.1) than the healthy group (3.6, IQR: 3.2–4.0). The small difference in Shannon index was not statistically significant (Wilcoxon rank-sum test, $p > 0.05$), indicating that overall within-sample microbial diversity was broadly comparable between the two groups.

**Fig 9: Shannon diversity index comparing healthy and mixed *P. falciparum* and *P. malariae* infections**

# Beta diversity

Between study group differences was determined using beta-diversity depicted with the Principal Component Analysis (PCA) plot, Fig 10. There was partial separation between malaria and healthy participants. Malaria samples clustered more closely and indicates a more uniform biological profiles associated with infection. The healthy individuals displayed greater variability. Although some overlap occurred between groups, a general trend of divergence along PC1 suggests malaria-related shifts.

**Fig 10: PCA plot of beta-diversity**

Ellipses represent 95% confidence regions for each group. Axes/Legend labels: x-axis: PC1 (10.7% variance explained); y-axis: PC2 (8.4% variance explained); Legend: Health status (Healthy | Malaria).

# Discussion

Asymptomatic *Plasmodium* reservoirs perpetuate the transmission of malaria, making malaria control and eradication strategies virtually impossible (12). This study focused on how this "cluster of silent parasites" affected microbiome dynamics in children in the Ahanta West, Ghana. The results provide insights into how *Pl~~asmodium~~ falciparum* only and mixed (*P. falciparum* and *P. malariae*) infections influence gut microbial composition and diversity. The higher prevalence of mixed infections (*P. falciparum* and *P. malariae*) compared to single *P. falciparum* infections is consistent with reports from other studies conducted in sub-Saharan Africa, where mixed-species infections are common in endemic settings (11). According to Boundenga *et al.* (2024), mixed infections can lead to severe malaria during the asymptomatic to symptomatic transition due to interspecies interaction, followed by increased parasitaemia. In addition, non-*falciparum* species have been found to cause severe anaemia and renal disease in adolescents; therefore, the reservoir of mixed species in these asymptomatic carriers is a significant risk factor for complicated malaria (13).

The gut microbiome plays a central role in modulating host immunity and shaping disease outcomes through microbial–immune interactions (2,3). Our findings thus reveal changes in microbial diversity and composition in children with asymptomatic malaria infections compared to healthy controls. Among the twelve microbial phyla identified, Firmicutes and Bacteroidetes constitute nearly 70% of the total microbial population. This distribution aligns with findings from previous microbiome studies in both healthy and malaria-infected individuals (4). However, a shift in microbial abundance was observed such that Firmicutes, which were more abundant in healthy controls, showed a decrease in asymptomatic malaria cases, whereas Bacteroidetes increased. This shift is significant, as a reduction in Firmicutes has been associated with increased inflammation and altered gut homeostasis in animal models (4). Thus, the observed increase in Proteobacteria

suggests inflammatory stress and microbial imbalance associated with asymptomatic malaria, consistent with prior reports (9).

Prevotella was the most abundant taxon at the genus level, followed by *Faecalibacterium*. While *Faecalibacterium* was abundant in both groups, its reduction in asymptomatic malaria-infected individuals is of concern, as this genus is a known producer of butyrate that has anti-inflammatory properties (14). The increase in *Alloprevotella* and *Streptococcus* in the asymptomatic malaria-infected group further highlights potential disruptions in microbial stability, as these genera have been associated with dysbiosis in other diseases (15,16).

The children with mixed infections exhibited microbiome alterations similar to those observed in *P. falciparum*-only infections but with notable differences. The observed increase in *Escherichia-Shigella* in mixed infections is noteworthy, as this genus includes pathogenic species that contribute to gut inflammation and increased susceptibility to enteric infections (17)

Alpha-diversity analysis showed no significant differences in microbial richness and evenness between groups, suggesting that overall gut diversity remains relatively stable during asymptomatic infection. However, beta-diversity analysis revealed distinct microbial community structures associated with malaria status, indicating compositional shifts rather than broad loss of diversity. This is evident as malaria status accounted for more than 10% of the variation observed in the gut microbiome. These findings suggest a potential link between *Plasmodium* infection and gut microbiota alterations and highlight microbial shifts associated with disease status. While the overall diversity may not change, malaria infection significantly alters the specific composition of the gut microbiome. Future longitudinal studies that integrate metagenomics and metabolomics will be required to determine whether these compositional shifts drive susceptibility to symptomatic malaria or alter treatment response.

# Conclusion

This study provides novel insights into the gut microbiome alterations associated with asymptomatic malaria infections. The observed decrease in Firmicutes and Faecalibacterium, alongside an increase in Bacteroidetes, Proteobacteria, and Escherichia-Shigella, suggests a disruption in gut microbial homeostasis. Mixed infections appear to amplify gut microbial disruption, indicating synergistic effects on host-microbe balance. Understanding these microbial signatures may guide microbiome-targeted adjunct therapies and support malaria surveillance in endemic communities.

# Acknowledgements

The authors gratefully acknowledge the microbiota vault for their services provided. AAL is supported by a grant from the World Academy of Sciences (TWAS) (Grant Number: 21-111 RG/BIO/AF/AC_1-FR3240319478). The funder had no role in study design, data collection and analysis, decision to publish, or preparation of the manuscript.

# References


1. Bousema T, Okell L, Felger I, Drakeley C. Asymptomatic malaria infections: detectability, transmissibility and public health relevance. Nat Rev Microbiol. 2014 Dec;12(12):833–40.

2. Pickard JM, Zeng MY, Caruso R, Núñez G. Gut microbiota: Role in pathogen colonization, immune responses, and inflammatory disease. Immunol Rev. 2017 Sep;279(1):70–89.

3. Yoo J, Groer M, Dutra S, Sarkar A, McSkimming D. Gut Microbiota and Immune System Interactions. Microorganisms. 2020 Oct 15;8(10):1587.

4. Villarino NF, LeCleir GR, Denny JE, Dearth SP, Harding CL, Sloan SS, et al. Composition of the gut microbiota modulates the severity of malaria. Proc Natl Acad Sci. 2016 Feb 23;113(8):2235–40.

5. Fan Y, Pedersen O. Gut microbiota in human metabolic health and disease. Nat Rev Microbiol. 2021 Jan;19(1):55–71.

6. Lobionda S, Sittipo P, Kwon HY, Lee YK. The Role of Gut Microbiota in Intestinal Inflammation with Respect to Diet and Extrinsic Stressors. Microorganisms. 2019 Aug 19;7(8):271.

7. Saltzman ET, Palacios T, Thomsen M, Vitetta L. Intestinal Microbiome Shifts, Dysbiosis, Inflammation, and Non-alcoholic Fatty Liver Disease. Front Microbiol. 2018 Jan 30;9:61.

8. Zhao L. The gut microbiota and obesity: from correlation to causality. Nat Rev Microbiol. 2013 Sep;11(9):639–47.

9. Fukui H. Role of Gut Dysbiosis in Liver Diseases: What Have We Learned So Far? Diseases. 2019 Nov 12;7(4):58.

10. Portincasa P, Bonfrate L, Vacca M, De Angelis M, Farella I, Lanza E, et al. Gut Microbiota and Short Chain Fatty Acids: Implications in Glucose Homeostasis. Int J Mol Sci. 2022 Jan 20;23(3):1105.

11. Abdulraheem MA, Ernest M, Ugwuanyi I, Abkallo HM, Nishikawa S, Adeleke M, et al. High prevalence of Plasmodium malariae and Plasmodium ovale in co-infections with Plasmodium falciparum in asymptomatic malaria parasite carriers in southwestern Nigeria. Int J Parasitol. 2022;52(1):23–33.

12. Cheaveau J, Mogollon DC, Mohon MAN, Golassa L, Yewhalaw D, Pillai DR. Asymptomatic malaria in the clinical and public health context. Expert Rev Anti Infect Ther. 2019 Dec 2;17(12):997–1010.

13. Boundenga L, Sima-Biyang YV, Longo-Pendy NM, Bignoumba M, Mbou-Boutambe C, Moukagni-Mussadji CJ, et al. Epidemiology and diversity of Plasmodium species in Franceville and their implications for malaria control. Sci Rep. 2024;14(1):31977.

14. Vesterbacka J. The role of microbial translocation and gut microbiota in HIV-1 infection [Internet]. Karolinska Institutet (Sweden); 2017 [cited 2025 Mar 25]. Available from: https://search.proquest.com/openview/7c19815c7ce455a239ac69ad22bab681/1?pq-origsite=gscholar&cbl=2026366&diss=y

15. Gu Z, Zhang Y, Zhao X, Liu T, Sheng S, Song R, et al. Comparing sputum microbiota characteristics between severe and critically ill influenza patients. Front Cell Infect Microbiol. 2023 Dec 18;13:1297946.

16. Liu Y, Wang H, Jiang H, Sun Z, Sun A. Alloprevotella Can be Considered as a Potential Oral Biomarker in Intestinal Metaphase of Gastric Patients. In: Advances in Biomedical and

Bioinformatics Engineering [Internet]. IOS Press; 2023 [cited 2025 Mar 25]. p. 155–67. Available from: https://ebooks.iospress.nl/doi/10.3233/SHTI230836

17. Strockbine NA, Bopp CA, Fields PI, Kaper JB, Nataro JP. *Escherichia* , *Shigella* , and *Salmonella*. In: Jorgensen JH, Carroll KC, Funke G, Pfaller MA, Landry ML, Richter SS, et al., editors. Manual of Clinical Microbiology [Internet]. Washington, DC, USA: ASM Press; 2015 [cited 2025 Mar 25]. p. 685–713. Available from: http://doi.wiley.com/10.1128/9781555817381.ch37

Figure 1

Click here to access/download;Figure;Fig 1.tif

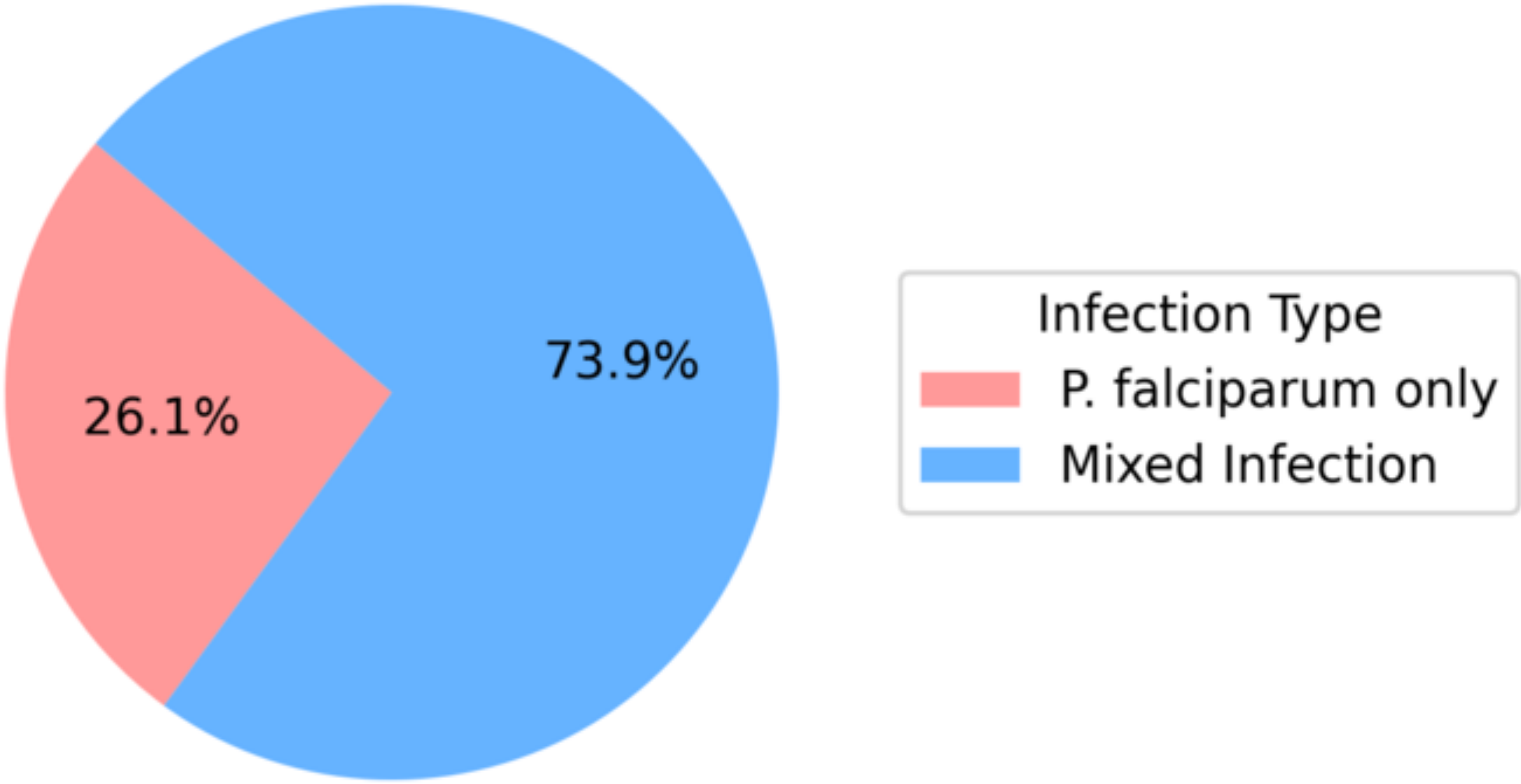

Figure 2

Click here to access/download;Figure;Fig 2.tif

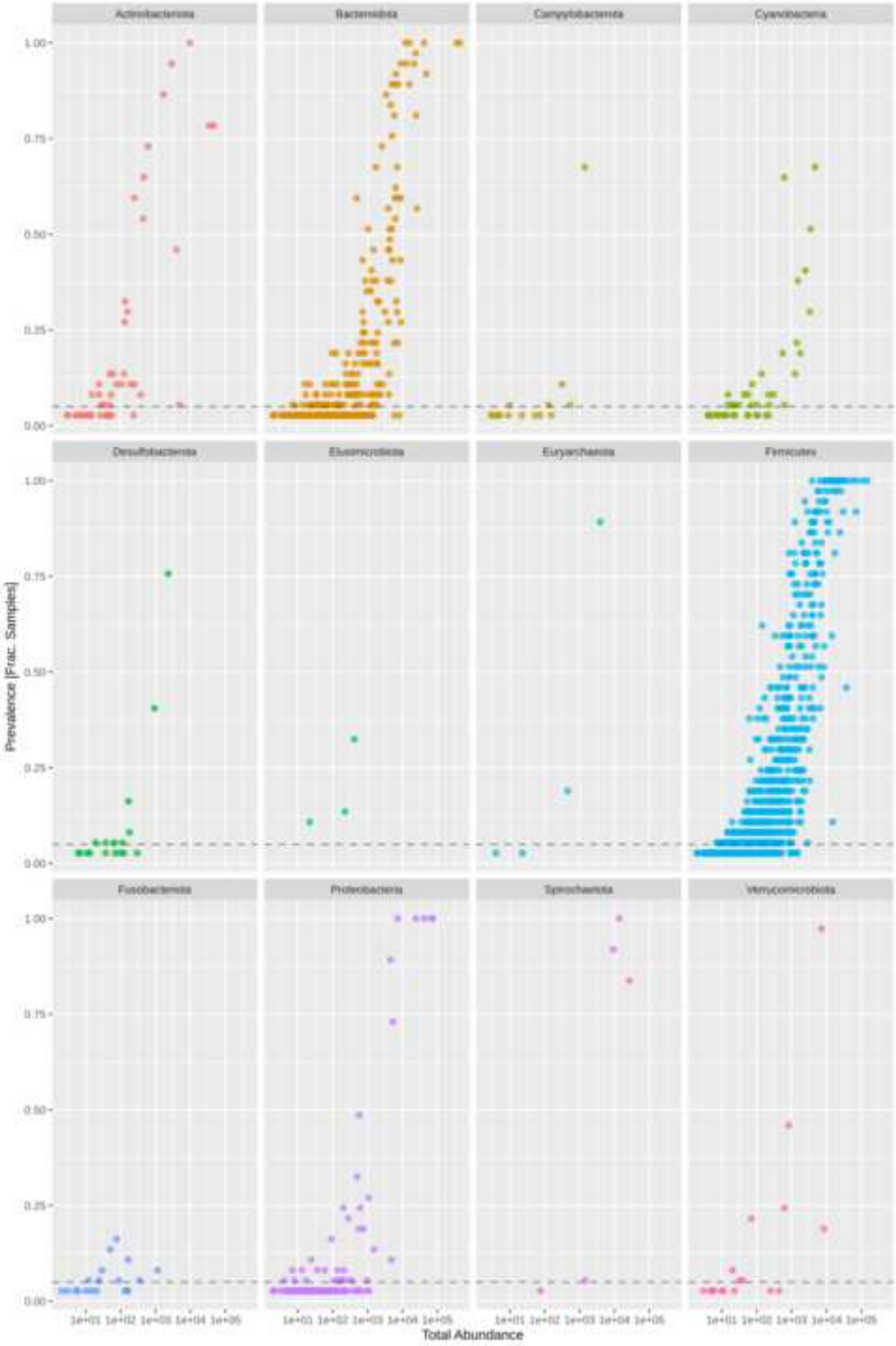

Figure 3

Click here to access/download;Figure;Fig 3.tif

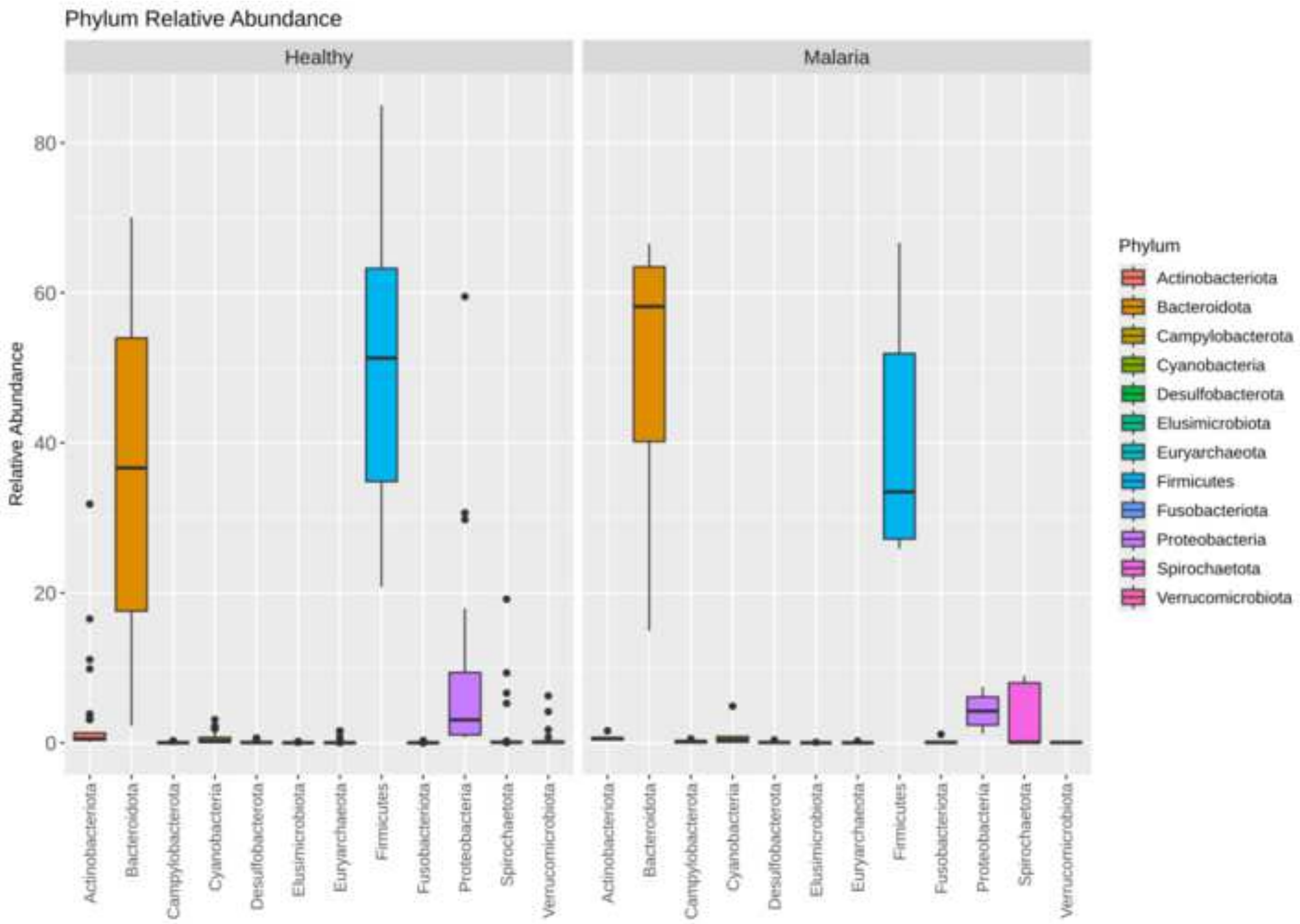

Figure 4

Click here to access/download;Figure;Fig 4.tif

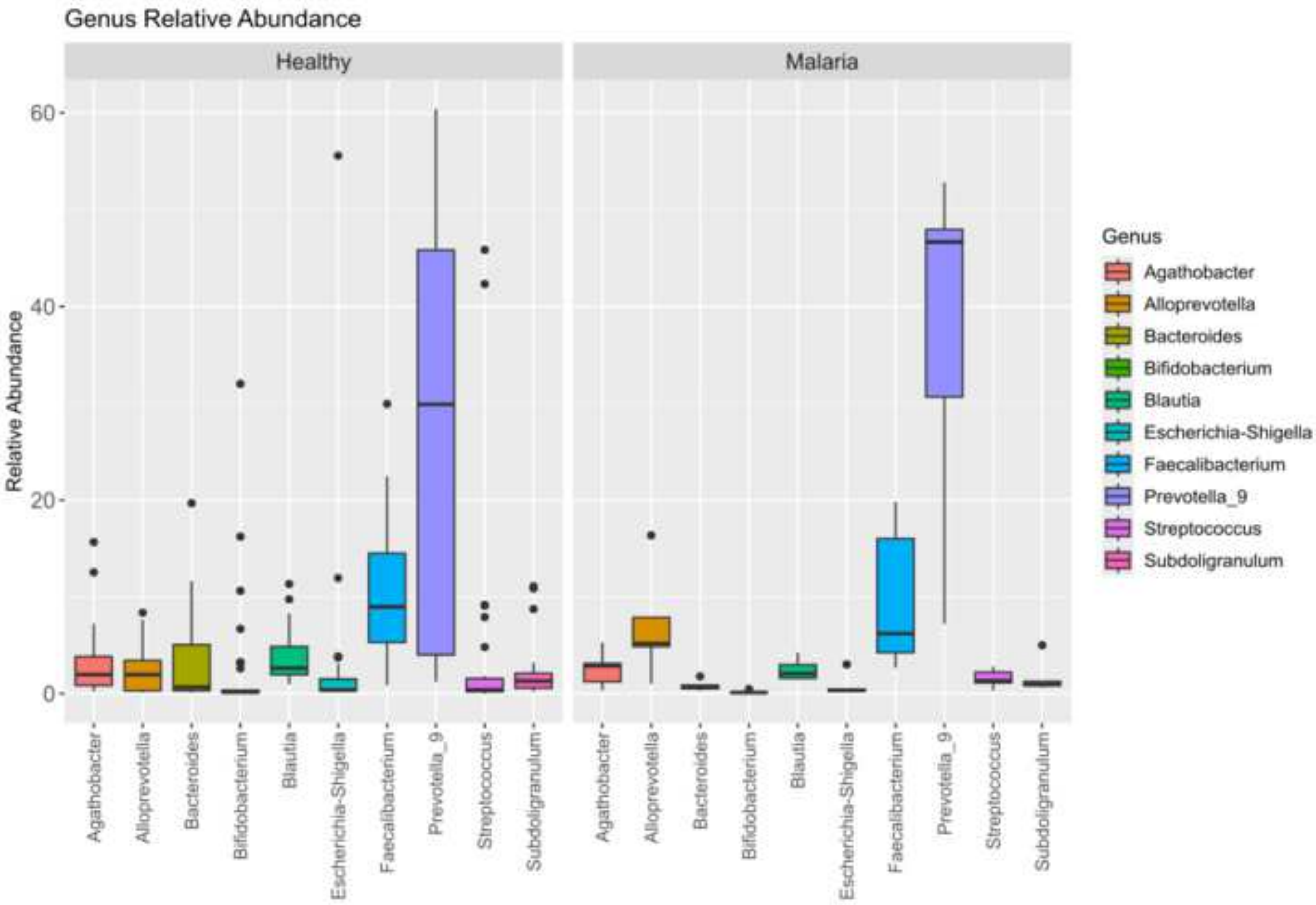

Figure 5

Click here to access/download;Figure;Fig 5.tif

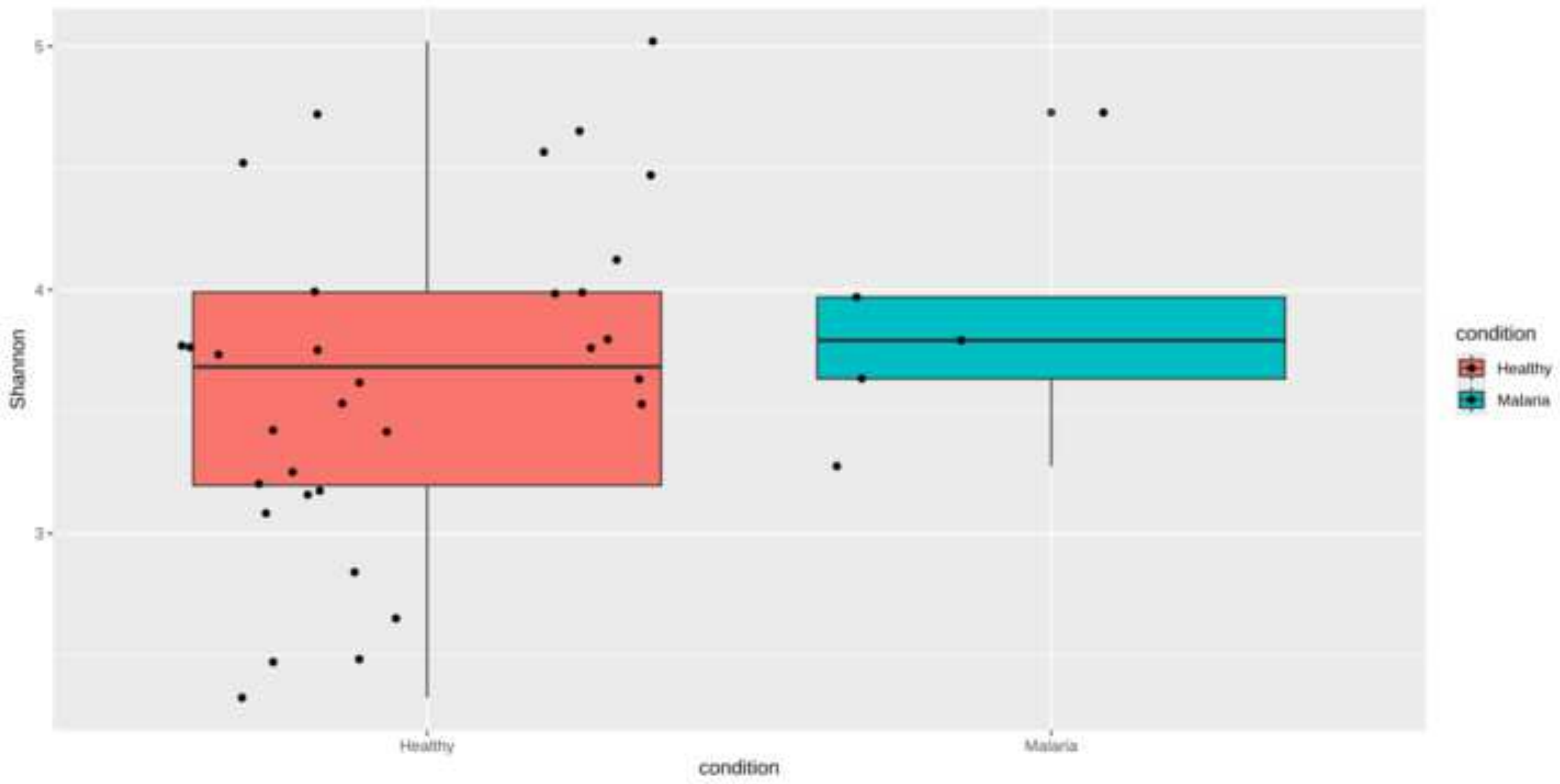

Figure 6

Click here to access/download;Figure;Fig 6.tif

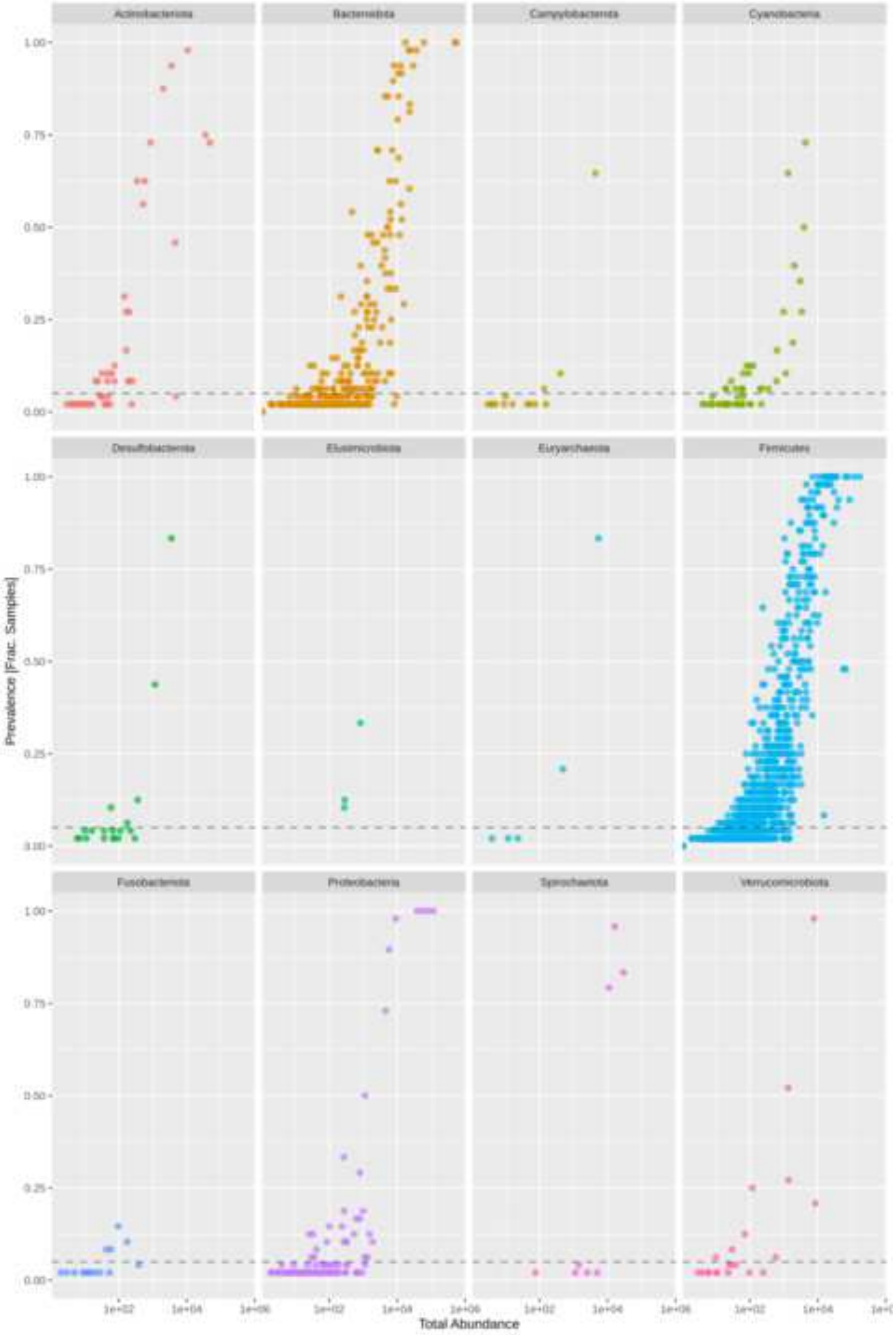

Figure 7

Click here to access/download;Figure;Fig 7.tif

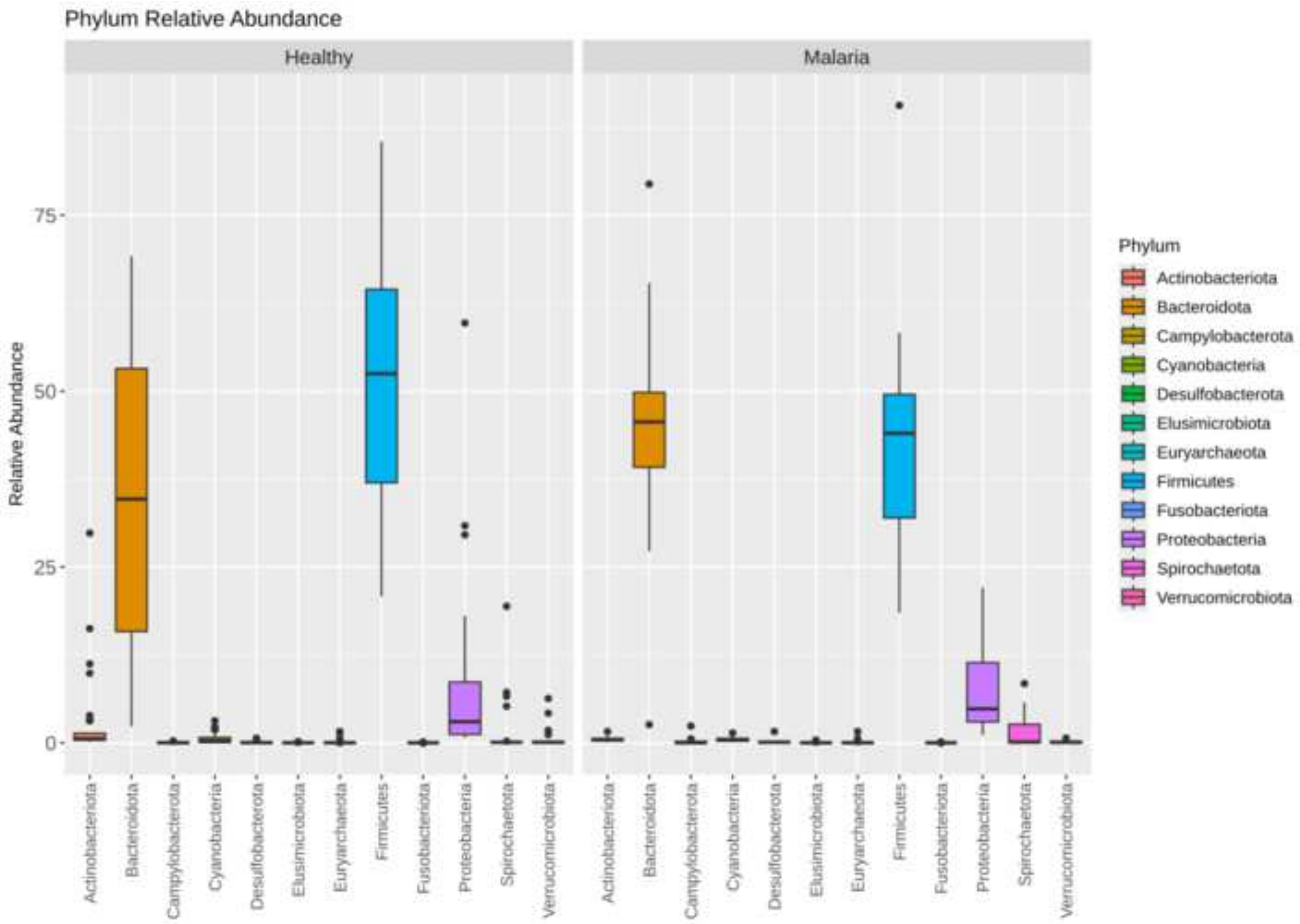

Figure 8

Click here to access/download;Figure;Fig 8.tif

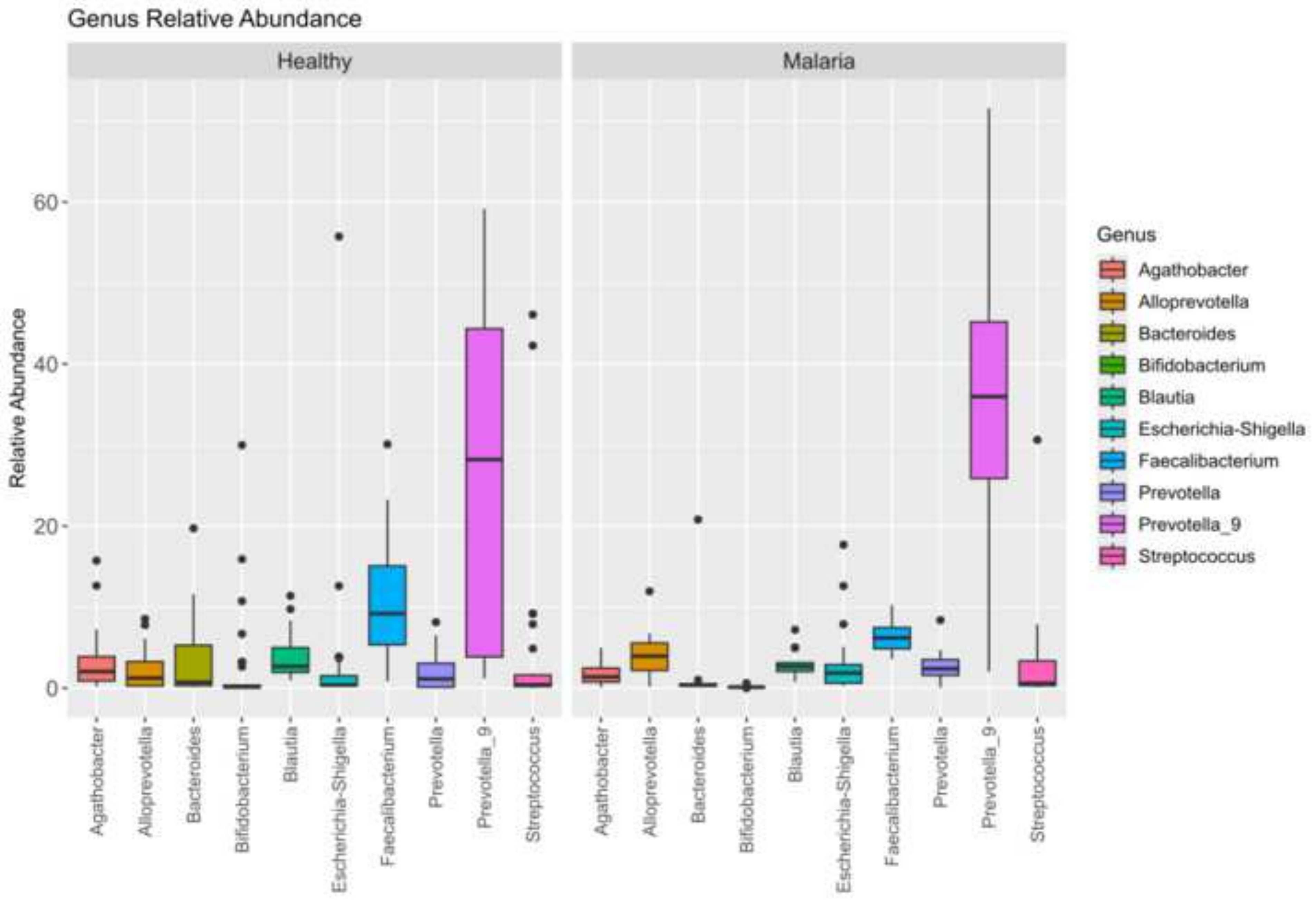

Figure 9

Click here to access/download;Figure;Fig 9.tif

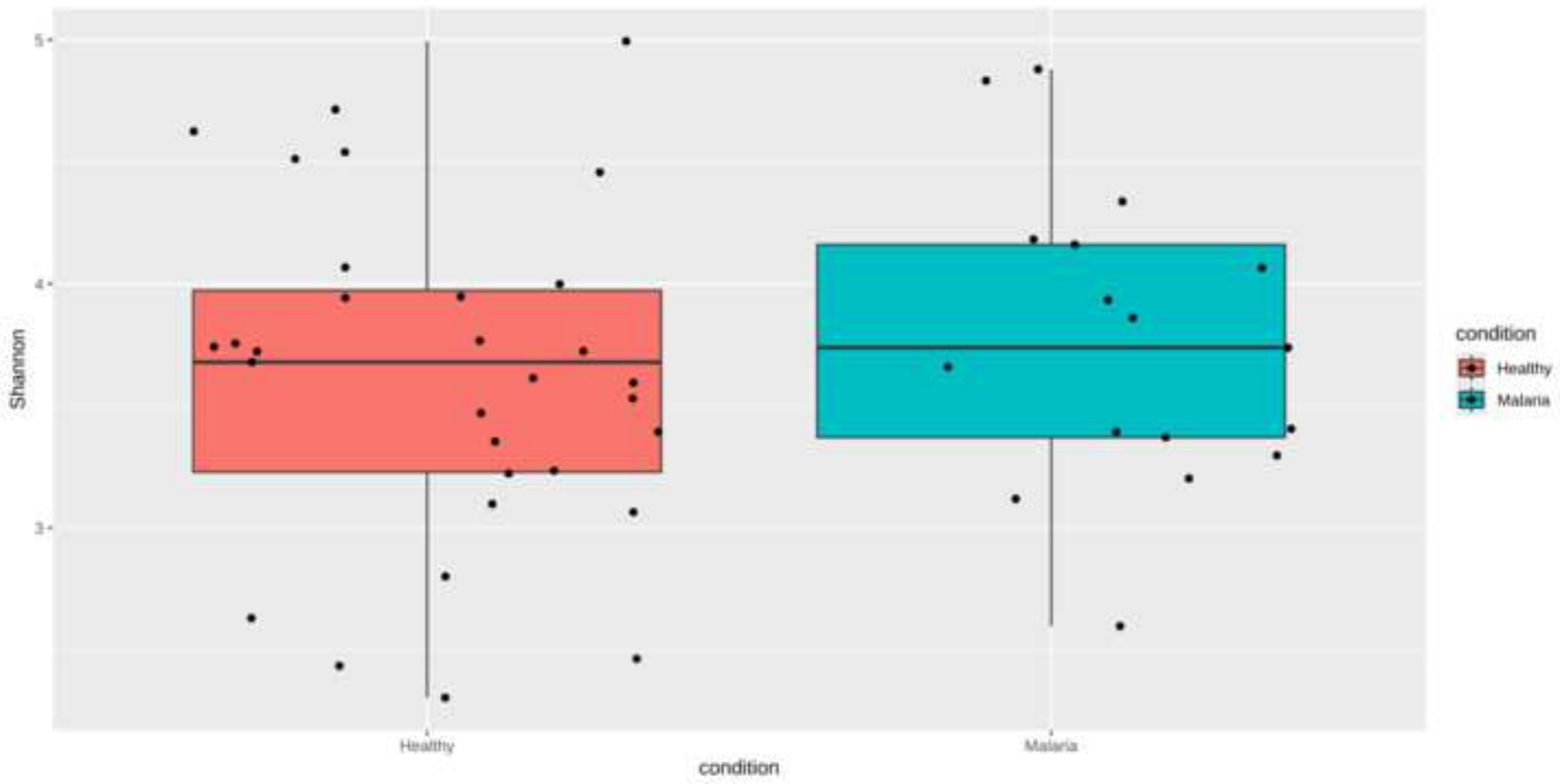

Figure 10

Click here to access/download;Figure;Fig 10.tif

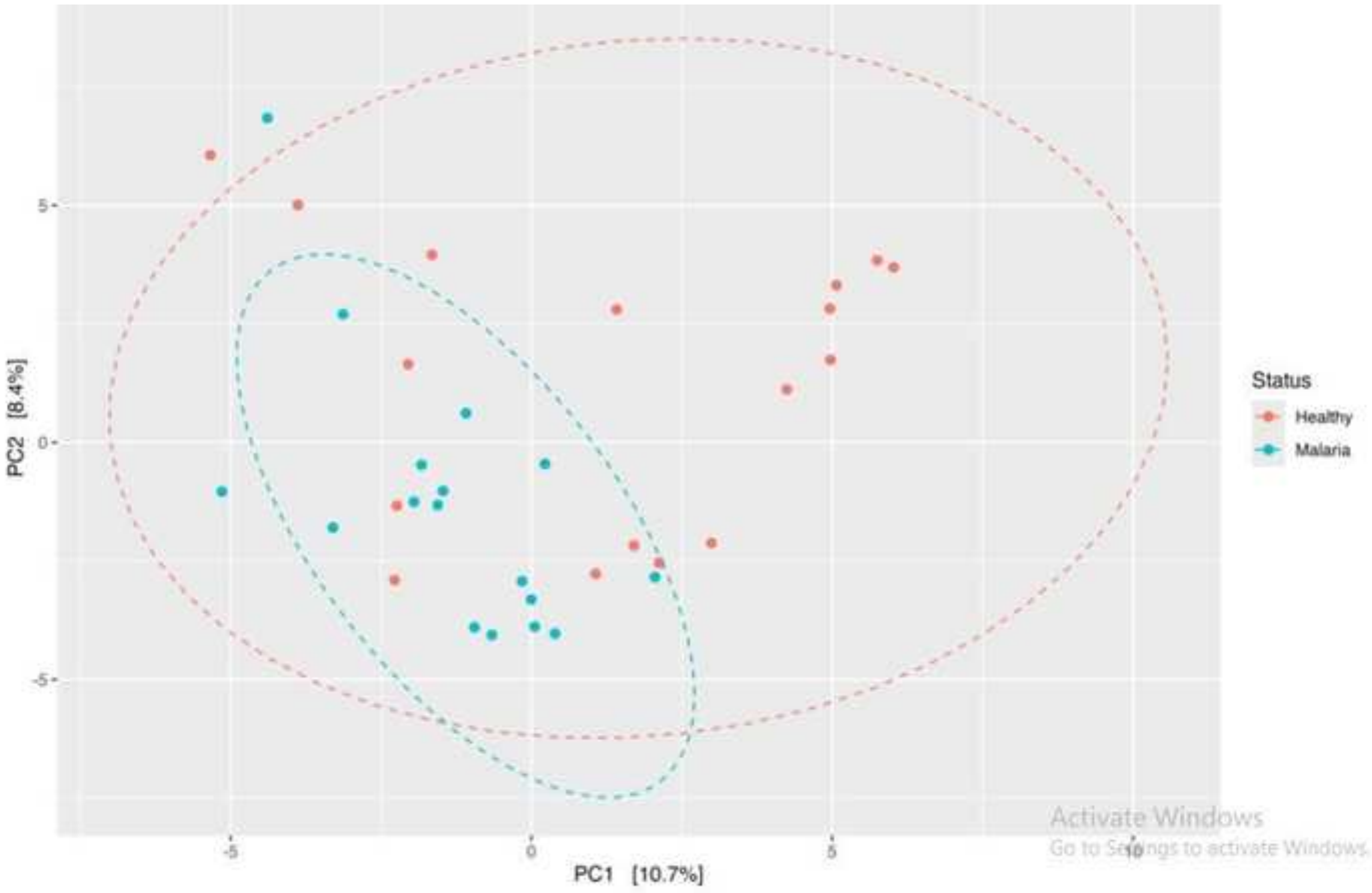


Activate Windows
Go to Settings to activate Windows.
