# Supplementary material for: Gut microbiome alterations among Ghanaian children with asymptomatic malaria infections
Source: PLoS One. 2026 Apr 24;21(4):e0348120. doi: 10.1371/journal.pone.0348120 (PMC13108879; doi:10.1371/journal.pone.0348120)
Supplement: Attachment — Submitted filename: Response to Reviewers.docx [file pone.0348120.s011.docx]

Reviewer #1

Comment:

The manuscript addresses a relevant topic and presents useful data. However, revision is required to improve flow and clarity. Scientific names are inconsistently italicised, the results section needs restructuring, and the discussion should better focus on interpreting the findings. Please see my comments in the manuscript.

Response:

We have revised the manuscript with scientific names now consistently italicised throughout the text. The Results section has been reorganised for improved logical progression. All specific comments in the manuscript have been addressed accordingly.

Reviewer #2

In general, it is important to note that the article does not indicate how many of the 113 recruited children have asymptomatic malaria and how many do not. This information is fundamental for evaluating the comparison. There is no table comparing the two groups and their epidemiological characteristics. Providing only the overall epidemiological variables shown in Table 1 is insufficient.

Response:

We have now clearly stated the proportion of children with asymptomatic malaria (PCR-positive, n = 86) and those without infection (PCR-negative, n = 27) in the Results section. In addition, we have included a new table comparing key demographic characteristics (age, height, weight, and BMI-for-age Z-scores) between the two groups, along with corresponding statistical significant levels.

Comment 1:

Distribution of other chronic infections

The manuscript does not report on the prevalence of other infections that could have a significant influence on chronic inflammatory status and the composition of the gut microbiota, such as HIV infection or hepatotropic viruses. These are important potential confounders. The distribution of such infections across groups should be reported and controlled for in the analysis.

Response:

Participants with known self-reported chronic infections were excluded from the microbiome analysis to minimize potential confounding effects. This has now been added to the Methods section.

Comment 2:

Nutritional status of participants

Nutritional status is a major determinant of gut microbiome composition and immune function. The authors should provide anthropometric data, at least BMI. It should also be clarified whether the distribution of BMI differed between groups. The reported mean BMI of 16.3 suggests that many children may be underweight. A more detailed characterisation of nutritional status is necessary to determine whether the groups are truly comparable.

Response:

BMI-for-age Z-scores (BMIA-Z) has been calculated for all participants. The mean BMIA-Z was −0.98 ± 0.92 in PCR-negative children and −1.03 ± 0.87 in PCR-positive children, with no significant difference between groups. These are now in the Results section in characteristics table.

Comment 3:

Dietary assessment

Dietary intake strongly influences microbiome composition. The absence of dietary data is a significant limitation. If such data were collected, they should be included. Otherwise, this limitation should be clearly acknowledged and discussed.

Response:

We agree that dietary intake strongly influences gut microbiome composition. Dietary data were not collected in this study, which limits our ability to assess its contribution. We have now explicitly acknowledged this limitation in the Discussion section.

Comment 4:

Intestinal parasites

The authors should explain why children infected with intestinal parasites were not excluded from the study. Such infections can directly affect the composition of the gut microbiota and may distort the results.

Response:

All children were screened for intestinal parasites. Participants found to be infected were excluded from the microbiome analysis. It has been added as exclusion criterion in the Methods section.

Comment 5:

Post-treatment microbiome assessment

A post-treatment microbiome analysis would strengthen causal inference by demonstrating whether microbiome alterations are reversed after parasite clearance. While this may not be feasible retrospectively, this should be discussed as a limitation

Response:

We agree that longitudinal follow-up after parasite clearance would strengthen causal inference. However, such sampling was not collected for this study. We have acknowledged this as a limitation in the Discussion and suggested it as an important direction for future research.

Comment 6:

Comparisons with symptomatic malaria

Including or discussing comparisons with children with symptomatic malaria would have provided important context and helped determine whether the observed alterations are specific to asymptomatic infection.

Response:

Our study recruited children with asymptomatic malaria, and symptomatic cases were not included in this cohort. We have now acknowledged this as a limitation in the Discussion section and noted that inclusion of symptomatic cases in future studies would help clarify whether observed microbiome alterations are specific to asymptomatic infection.
