# Supplementary material for: Gut microbiome alterations among Ghanaian children with asymptomatic malaria infections
Source: PLoS One. 2026 Apr 24;21(4):e0348120. doi: 10.1371/journal.pone.0348120 (PMC13108879; doi:10.1371/journal.pone.0348120)
Supplement: Attachment — Submitted filename: Response_to_Reviewers_auresp_2.docx [file pone.0348120.s012.docx]

Reviewer #2

One important aspect remains insufficiently addressed. Specifically, the potential inclusion of children with chronic diseases that may influence systemic inflammation and consequently the microbiota has not been clearly specified. Furthermore, the absence of a screening process for such conditions, particularly those that may be asymptomatic or undiagnosed, is a limitation as their potential impact cannot be ruled out. This should be explicitly acknowledged in the discussion as it is relevant to the interpretation of the results and constitutes an important limitation of the study.

Response:

We appreciate this important comment. Participants with known self-reported chronic conditions that that may influence the study outcome were excluded from the study. However, we acknowledge that this approach may not fully account for undiagnosed or asymptomatic chronic conditions. We have now included this as a limitation in the Discussion section as recommended.
